# Senescence in human AC16 cardiac cells is associated with thymidine kinase induction and histone loss

**DOI:** 10.17912/micropub.biology.000865

**Published:** 2023-06-29

**Authors:** Nikhitha Kastury, Veronica Hidalgo, Boomathi Pandi, Lauren Li, Maggie P. Y. Lam, Edward Lau

**Affiliations:** 1 Medicine, University of Colorado Anschutz Medical Campus, Aurora, Colorado, United States

## Abstract

AC16 cells are a transformed human cardiac cell line commonly used to study cardiomyocyte biology. We show that reduced proliferation and senescence markers can be robustly induced in AC16 cells cultured in low serum condition and treated with (i) low-dose doxorubicin, (ii) UV 254 nm, or (iii) H _2_ O _2_ exposure for up to 48 hours. Increased p21 (CDKN1A) and H2A.X variant histone (H2AX) levels serve as reliable molecular markers upon all three treatment conditions, but the up-regulation of another common senescence marker, p16 (CDKN2A) was not observed. A proteomics screen further shows that the loss of histones and the increased expression of thymidine kinases (TK1) are prominent features of AC16 cells under doxorubicin induced senescence.

**Figure 1. Induction and characterization of senescence in human AC16 cardiac cells.**
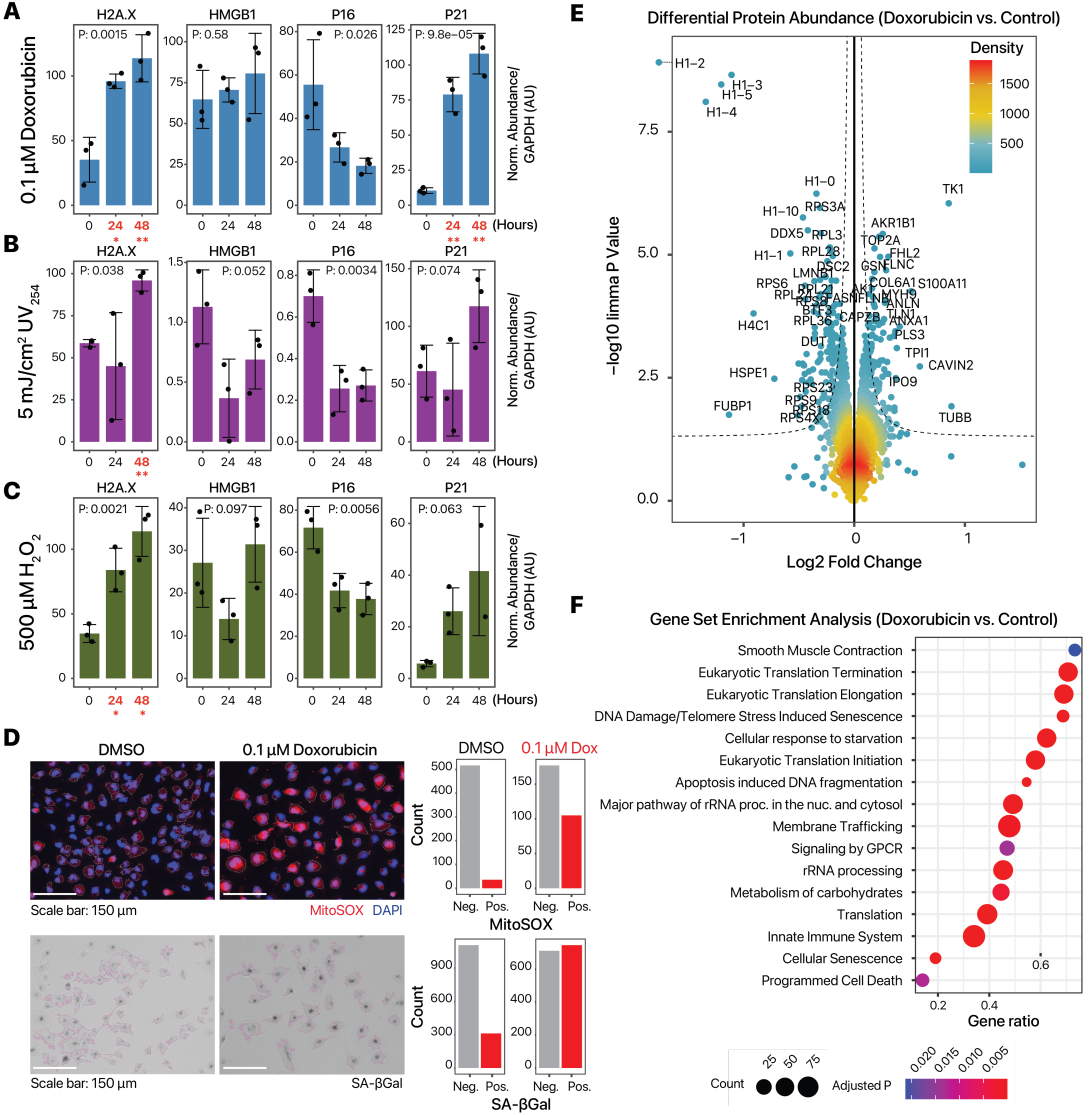
**A–C.** Densitometry of immunoblots showing induction of H2A.X (H2AX) and p21 (CDKN1A) after treatment with 0.1 µM doxorubicin, 5 mJ/cm ^2^ 254 nm UV, and 500 µM H _2_ O _2_ up to 48 hours. P values: ANOVA, n=3 per condition, except for P21 48 hours H _2_ O _2_ , where n=2 due to an incomplete blot. Treatment conditions that are significantly increased from 0 hour are in red and bold (*: two-tailed unpaired t-test P < 0.05; **: t-test P < 0.01 vs. 0 hour). **D.** MitoSOX staining (top) and senescence associated beta-galactosidase (SA-βGal) staining (bottom) after 24 hours of 0.1 µM doxorubicin (Dox) corroborates induction of senescence. Bar charts show the number of MitoSOX or SA-βGal positive cells in 0.1 µM doxorubicin vs. DMSO vehicle Scale bar: 150 µm. In the MitoSOX staining: white outline: identified cell objects; blue outline; identified nuclei; blue: DAPI. In the SA-βGa staining, magenta outline: identified cell objects. **E.** Volcano plot of tandem mass tag labeled bottom-up proteomics experiments, showing the significant induction of thymidine kinase (TK1) and suppression of multiple histones (H1-2, H1-3, H1-4, H1-5). X-axis: log2 fold change (doxorubicin vs. DMSO). Y-axis: –log10 of limma P value (n=4). Color denotes number of data point neighbors. **F.** Gene set enrichment analysis of protein quantification (doxorubicin vs. DMSO) showing a number of significantly enriched terms implicated in AC16 senescence. Size denotes number of quantified proteins in the gene set, color: GSEA FDR adjusted P value.

## Description

Human AC16 cardiac cells are a transformed cell line made from fusing cardiomyocytes with cardiac fibroblast and transforming with SV40 (Davidson et al., 2005) . Since its original description in 2005, this cell line has seen increasingly common use in cardiac biology for modeling human cardiomyocyte biology and disease mechanisms (Onódi et al., 2022). Cellular senescence refers to the process whereby cells have reached their replicative capacity and enter a non-replicative state. Although adult cardiomyocytes are thought to have already exited the cell cycles, induction of premature senescence in response to cardiotoxic insults such as oxidative stress or DNA damage can lead to many cardiac pathologies that are relevant to cardiac aging (Gude et al., 2018) . As such, there is interest in using AC16and other cardiac cell lines to model senescence and aging in vitro. The induction of senescence is often recognized in various cell types using markers that are activated in senescent cells including cell cycle regulator proteins p53 (TP53), p21 (CDKN1A) or p16/INK4a (CDKN2A) (Kumari & Jat, 2021) . However, whether these markers are active in human AC16 line and reliable methods for inducing senescence are not widely established.

We assessed whether cellular senescence markers can be reliably introduced by different insults in AC16 cells as a cardiac aging model. We found three successful conditions, where human AC16 cells were continually exposed to 0.1 µM of doxorubicin or 500 µM H _2_ O _2_ , or exposed to a single dose of 5 mJ/cm ^2^ 254 nm UV (UV-C) light, then cultured in low serum (1% FBS) condition for 24 hours and 48 hours. UV-C induces DNA damage in cultured cells. Doxorubicin is a genotoxic cancer drug that is associated with cardiac toxicity and at low dose has been thought to induce cardiac aging whereas H _2_ O _2_ introduces oxidative stress, both of which are commonly used for cellular senescence (Piegari et al., 2013; Spallarossa et al., 2009) . Each condition led to an observable decrease in proliferation rates of the cells. Moreover, the effect of these insults on the expression of several classical senescence markers was tested by immunoblotting ( **Figure** **1A–C** ). We found that the pro-senescence conditions induced p21 (CDKN1A) and H2A.X variant histone (H2AX) as expected. The induction was the most robust in doxorubicin, followed by H _2_ O _2_ exposure. Unexpectedly, the common senescence marker p16 (CDKN2A) showed a decrease rather than increase in expression following exposure, suggesting it may not be a useful marker for AC16 cells under the tested treatment doses and duration. There are also no overall changes in the total level of high mobility group box 1 (HMGB1), which migrates from the nucleus to the cytosol in senescent cells.

As 0.1 µM doxorubicin induced senescence markers most reliably, we characterized additional cellular and molecular changes in this condition. Addition of 0.1 µM doxorubicin reliably induced mitochondrial superoxide formation as evidenced in MitoSOX staining ( **Figure 1D** ). We next examined the proteomic changes in senescence using mass spectrometry based bottom-up proteomics. AC16 cells were treated with or without doxorubicin for 24 hours as above, then co-cultured with fibroblasts for 24 hours as part of a larger study. The cells were then harvested for mass spectrometry. In total, we quantified the expression of 2,857 proteins in AC16 cells with or without doxorubicin treatment (n=4 each) ( **Figure 1E** ), with 279 proteins differentially regulated at 5% FDR (61 at 1% FDR). Among the most significantly differentially regulated proteins, we find an upregulation of thymidine kinase (TK1) (logFC: 0.85; adj. P: 4.3e–4) and a down regulation of multiple histone proteins, including histone H1.2 (H1F2) (logFC: –1.77; limma adjusted P: 3.2e–6), histone H1.3 (H1F3) (logFC: –1.1; limma adjusted P: 3.2e–6), and histone H1.5 (H1F5) (logFC: –1.20; limma adjusted P: 3.3e–6). Consistent with the immunoblot results, no significant change was observed in the CDKN2A protein whose locus encodes p16 (logFC –0.03; limma adjusted P: 0.20).

Gene set enrichment analysis (GSEA) of the proteomics data set highlights the enrichment of several classes of related gene sets, including terms related to Translation (GSEA adjusted P: <2.8e–19), and DNA Damage/Telomerase Stress Induced Senescence (EnrichmentScore –0.86: GSEA adjusted P: <1.4e–4) ( **Figure 1F** ). The Translation related terms are driven by a decreased abundance of ribosomal proteins, including RPS3A (logFC: –0.31; limma adjusted P: 4.5e–4), RPL3 (logFC: –0.30; limma adjusted P: 9.8e–4), RPS11 (logFC: –0.22; limma adjusted P: 1.5e–3), RPL28, RPL4, RPL34, RPS6, and others. The senescence terms are driven by histone chaperone ASF1A (logFC: –0.13; limma adjusted P: 0.07), lamin B1 (LMNB1) (logFC: –0.31; limma adjusted P: 3.5e–3), p53 (TP53) (logFC: –0.14; limma adjusted P: 8.6–3), and others. Hence overall, doxorubicin robustly induces senescence-related pathways including a reduction of ribosomal proteins, and a depletion of lamin B1 (LMNB1) and p53 (TP53).

In summary, we find that senescence markers p21 and H2A.X can be reliably induced using multiple experimental perturbations. The robust induction of p21 in the three DNA-damage related senescence models tested is notable as it may be directly related to senescence associated secretory phenotypes (Englund et al., 2023; Sturmlechner et al., 2021) . An unbiased proteomics experiment further shows that AC16 senescence is associated with reduced ribosomal level, and senescence pathways including lamin B1 (LMNB1) and p53 (TP53), the latter of which is upstream of p21 in relaying DNA damage to senescence maintenance. A global loss of histones is also observed, which is generally seen in aging and is thought to contribute to the loss of chromatin structure (Sen et al., 2016; Singh et al., 2019) . Finally, thymidine kinase 1 (TK1) is robustly induced, a protein that is responsible for generating the nucleic acid thymidine for DNA synthesis and repair. Unexpectedly, we find that p16 immunoblot signals are suppressed in human AC16 cells upon senescence treatment in our hands. Although p16 is one of the most established aging and senescence markers (Kim & Sharpless, 2006) , the relative roles of p53/p21 and p16 on senescence induction is known to depend on the type and duration of induced stress (Van Deursen, 2014). Overall, the models presented here may avail the ongoing studies of the effect of aging in the heart. Whether these insults affect different pathways or can be combined to induce senescence more efficiently would be a topic of interest. Future work can also apply these methods to other cardiac cell types used in in vitro experiments including H9c2 rat myocytes or human pluripotent stem cell derived cardiomyocytes.

## Methods

**Cell culture**

AC16 acquired from Millipore and cultured in DMEM/F12 with 10% FBS at 37 °C and 5% CO _2_ . Cells were used at passages 8 to 12. For immunoblots, the cells were switched to DMEM/F12 with 1% FBS and exposed to the treatment for 24 to 48 hours as described. Prior to the mass spectrometry experiment, the AC16 cells were switched to Fibroblast Growth Medium (FGM-3) with supplements (Promocell) then treated with 0.1 µM doxorubicin for 24 hours, then co-cultured through non-contact transwells (Corning) with primary human ventricular cardiac fibroblasts (Promocell) with or without TGF-β stimulation for 24 hours as part of a companion study. The competence for senescence induction with doxorubicin and the lack of spontaneous senescence of the AC16 cells in FGM3 and co-culture was verified using p21 immunoblots.

**Imaging**

Mitochondrial superoxide staining was performed using MitoSOX stain (Invitrogen) following the manufacturer’s protocol. Briefly, AC16 cells were treated with 0.1 µM doxorubicin or vehicle for 24 hours, followed by staining with 5 µM MitoSOX for 10 minutes at 37 °C and 5% CO _2_ , washed three times with DPBS and imaged at 20× on an EVOS M5000 microscope (Thermo Scientific). Senescence associated β-galactosidase (Sigma) staining was performed following the manufacturer's protocol. Briefly, AC16 cells were treated with 0.1 µM doxorubicin or vehicle for 24 hours, fixed overnight at 37 °C, then imaged at 20× on an EVOS M5000 microscope (Thermo Scientific). Images were analyzed using CellProfiler v.4.2.5 (McQuin et al., 2018) to normalize backgrounds, perform cell object identification, and quantify integrated intensity.

**Immunoblots**

AC16 cells were harvested and pelleted; proteins were extracted using RIPA buffer plus protease inhibitors (Thermo Halt) and sonicated (Bioruptor Pico). The extracted protein concentration was quantified using BCA assay; 30 µg of proteins were separated by gel electrophoresis on a 5–20% Mini-PROTEAN TGX precast gel (Bio-Rad) then transferred to a PVDF membrane and visualized using Ponceau S staining. The membranes were washed in TBS-T, blocked using 5% TBS-T milk, and probed with primary antibodies and secondary antibodies listed in the reagents table following manufacturer instructions. Band intensity was normalized against GAPDH for quantification.

**Mass spectrometry**

For mass spectrometry, only AC16 cells in the co-culture were harvested and pelleted for this study. Proteins were extracted using RIPA buffer plus protease inhibitors (Thermo Halt) and solubilized in a sonicator (Bioruptor Pico). The extracted protein concentration was quantified using BCA assay; 25 µg of proteins were reduced, alkylated, and digested with trypsin using a filter-assisted protocol on a Pierce 10 K MWCO column as previously described (Han et al., 2022) . The peptides were then labeled using Tandem Mass Tag (TMT) 10-plex reagents (Thermo), combined, and separated by high-pH reversed phase separation (Thermo), then analyzed on an Orbitrap HF mass spectrometer with typical settings as described (Han et al., 2022) . Mass spectra were searched using Comet v.2022_01 (Eng et al., 2015) against a UniProt SwissProt (UniProt Consortium, 2021) human database with appended contaminants (retrieved 2023-03-22 using Philosopher (da Veiga Leprevost et al., 2020) ; 42,435 forward entries). The search results were further post-processed using Percolator (crux v.4.1 distribution) (The et al., 2016) , accepting 1% FDR. and TMT intensity was extracted using pyTMT v.0.4.1 as described (Dostal et al., 2020) .

**Statistics**

Protein differential expression statistics were calculated using limma v.3.52.4 (Ritchie et al., 2015) in R v.4.2.1 to assess the effect of doxorubicin on AC16 while adjusting the effect of TGF-β on the co-cultured fibroblasts. An FDR adjusted P value of 0.05 or below is considered statistically significant. Gene set enrichment analysis against Reactome was performed using ReactomePA v.1.40.0 (Yu & He, 2016) in R v.4.2.1. Additional data analysis was performed in R v.4.2.1. For immunoblots, quantitative data were shown as the mean ± standard deviation. An ANOVA or t-test P value ≤ 0.05 is considered statistically significant.

**Data Availability**

Raw mass spectrometry data are available on ProteomeXchange under the accession PXD041722. Immunoblot images are available on figshare at https://doi.org/10.6084/m9.figshare.22677085.

## Reagents

**Table:** 

Reagent	Manufacturer/Catalog number	Remarks
Anti-p21 rabbit monoclonal antibody	Cell Signaling #2947	1:1000 dilution
Anti-p16 rabbit monoclonal antibody	Cell Signaling #92803	1:1000 dilution
Anti-H2A.X rabbit monoclonal antibody	Cell Signaling #9718	1:1000 dilution
Anti-HMGB1 rabbit monoclonal antibody	Cell Signaling #6893	1:1000 dilution
Anti-GAPDH rabbit monoclonal antibody	Cell Signaling #5174	1:1000 dilution
Anti-rabbit IgG HRP-linked	Cell Signaling #7074	1:1000 dilution
